# Transcriptional Control of Axon Guidance at Midline Structures

**DOI:** 10.3389/fcell.2022.840005

**Published:** 2022-02-21

**Authors:** Eloísa Herrera, Augusto Escalante

**Affiliations:** Instituto de Neurociencias, CSIC-UMH, Alicante, Spain

**Keywords:** neuron, growth cone, axon pathfinding, target, transcriptional regulation, circuits development

## Abstract

The development of the nervous system is a time-ordered and multi-stepped process that includes neurogenesis and neuronal specification, axonal navigation, and circuits assembly. During axonal navigation, the growth cone, a dynamic structure located at the tip of the axon, senses environmental signals that guide axons towards their final targets. The expression of a specific repertoire of receptors on the cell surface of the growth cone together with the activation of a set of intracellular transducing molecules, outlines the response of each axon to specific guidance cues. This collection of axon guidance molecules is defined by the transcriptome of the cell which, in turn, depends on transcriptional and epigenetic regulators that modify the structure and DNA accessibility to determine what genes will be expressed to elicit specific axonal behaviors. Studies focused on understanding how axons navigate intermediate targets, such as the floor plate of vertebrates or the mammalian optic chiasm, have largely contributed to our knowledge of how neurons wire together during development. In fact, investigations on axon navigation at these midline structures led to the identification of many of the currently known families of proteins that act as guidance cues and their corresponding receptors. Although the transcription factors and the regulatory mechanisms that control the expression of these molecules are not well understood, important advances have been made in recent years in this regard. Here we provide an updated overview on the current knowledge about the transcriptional control of axon guidance and the selection of trajectories at midline structures.

## Introduction

The survival of organisms relies on their ability to detect stimuli, process sensory information and generate adequate motor responses. These functions depend on the precise organization of neural networks that enable communication between cells in an efficient and accurate manner. These networks emerge during embryonic development when newly born neurons extend axons away from the cell body to navigate through the developing embryo in order to reach their final targets. The growth cone at the tip of the travelling axon is a specialized structure armed with a plethora of receptors that defines the response of the growing axon to the environmental cues and determines its direction. The existence of both commissural neurons that project to the opposite side of the brain and ipsilateral neurons that connect with targets in the same hemisphere, is essential for the distribution and integration of sensory information and the subsequent generation of coordinated motor responses in species with bilateral symmetry (Colamarino and Tessier-Lavigne, 1995). Intense research during the last few decades focused on how ipsilateral and contralateral axons behave at the midline in different species and contexts has lead to the identification of many families of cues, receptors, and signaling cascades involved in axon pathfinding. Post-transcriptional mechanisms such as the microRNA-dependent regulation of guidance receptors (Yang et al., 2018), the regulation of local translation in axons (Zhuang et al., 2019; Corradi and Baudet, 2020), the role of lipids in axon guidance (Guy and Kamiguchi, 2021), novel ways of presenting guidance proteins (Dominici et al., 2017; Moreno-Bravo et al., 2017; Varadarajan et al., 2017; Wu et al., 2019; Dorskind and Kolodkin, 2021), interactions between different families of receptors (Zelina et al., 2014) or the targeted degradation of ligands or receptors (Gorla et al., 2019), all contribute to guarantee proper axon guidance progression and today we know that aberrant expression of axon guidance proteins or alterations in any of these mechanisms may result in a wide variety of neurodevelopmental diseases (Engle, 2010; Izzi and Charron, 2011; Nugent et al., 2012; Chédotal, 2014; Blockus and Chédotal, 2015; Van Battum et al., 2015; Roig-Puiggros et al., 2020). Despite these remarkable advances on the molecular mechanisms underlying axon guidance processes, current knowledge about the transcription factors (TFs) and the regulatory networks that orchestrate the expression of guidance molecules is still very limited (Butler and Tear, 2007). Here we provide an updated overview of the transcriptional mechanisms that control axonal trajectories during embryonic development paying particular attention to the navigation of neural axons at midline structures.

### Identification of Regulatory Factors Controlling Axon Guidance

Pioneer work on *Drosophila* initially identified a number of TFs involved in controlling the trajectories of motoneurons (MNs) axons towards their corresponding muscles and, subsequent work in vertebrates, revealed some of the transcriptional regulators that define specific limb muscles innervation (Landgraf et al., 1999; Keleman and Dickson, 2001; Dasen et al., 2003; Fujioka et al., 2003; Broihier et al., 2004; Dasen et al., 2005; Labrador et al., 2005; Garces and Thor, 2006; Layden et al., 2006). Further studies in vertebrates proposed that combinatorial codes of LIM proteins specify different MN trajectories and these TFs control the expression of specific axon guidance receptors from the EphA family to define MN trajectories to the different limb regions (Tsuchida et al., 1994; Sharma et al., 1998; Thor et al., 1999; Kania et al., 2000; Kania and Jessell, 2003; Shirasaki et al., 2006). In addition, another member of the homeobox TF family, Nkx2.9, was described to control the expression of the Slit receptor Robo2 and promote dorsal axon exit from the spinal cord in vertebrate spinal accessory MNs (Dillon et al., 2005; Bravo-Ambrosio et al., 2012).

Subsequently, other families of TFs have been associated with determining axonal trajectories in different neural circuits. For instance, the POU-domain TF Acj6 (abnormal chemosensory jump) was described as essential for the targeting of olfactory projection neurons in *Drosophila* (Komiyama et al., 2003), and Pou4f2, another member of the POU-family (aka Brn3.2 or Brn3b), seems to play an important role in the specification and pathfinding of retinal ganglion cell (RGC) axons (Erkman et al., 2000; Wang et al., 2000). Also in the visual system, members of the FOX family (FoxG1 and FoxD1) regulate the expression of the ephrinA receptors to determine the termination of retinal projections along the anterior-posterior axis at the visual targets (Herrera, 2004; Carreres et al., 2011). In the mouse cortex, the zinc-finger TFs Fezf2 and Ctip2 direct the projections of layer 5 corticospinal axons towards subcortical regions (Arlotta et al., 2005; Bin Chen et al., 2005; Jie-Guang Chen et al., 2005; Molyneaux et al., 2005; Lodato et al., 2014) and Ctip2 together with Satb2 control the formation of the corpus callosum (Srivatsa et al., 2014). In both vertebrates and invertebrates the Run-containing domain TFs control specific axonal trajectories since missexpression of Runt in *Drosophila* photoreceptors results in axons targeting the medulla instead of the lamina (Kaminker et al., 2002) and alterations in the levels of Runx3 shift the laminar termination of somatosensory neuron axons along the dorsoventral axis of the mouse spinal cord (Chen et al., 2006).

In addition to the abovementioned examples, two neuronal populations have been particularly useful to study the molecular mechanisms underlying axon pathfinding: spinal neurons at the time their axons navigate the floor plate, and retinal ganglion cells when their axons traverse the optic chiasm. In the following sections we review recent findings on the transcriptional regulation of neuronal trajectories using these two classic midline axon guidance models.

### Transcriptional Regulation of Axon Midline Crossing

The population of early born interneurons located in the most dorsal part of the spinal cord is known as dI1. As soon as dI1 neurons differentiate, they migrate ventrally to finally occupy the deep dorsal horns (Junge et al., 2016). A large number of reports studying this neuronal population have contributed to the current knowledge of how axons are attracted/repelled by guidance cues and their receptors [for recent reviews see (Chédotal, 2019; Comer et al., 2019)] and investigations on these neurons have also provided major insights into the regulatory mechanisms controlling axon guidance. There are two main subtypes of dI1 neurons: a population that occupies the medial intermediate spinal cord and project contralaterally (dI1c) and another cluster of cells that settle in the lateral intermediate spinal cord and avoid the floor plate to project ipsilaterally (dI1i). Both subtypes are derived from progenitor cells expressing the bHLH TF Atoh1 (Helms and Johnson, 1998, 2003; Lee et al., 1998; Helms et al., 2000; Gowan et al., 2001; Saba et al., 2005). Atoh1 induces the expression of the homeobox TFs Groucho co-repressors Barhl1 and Barhl2 (Bermingham et al., 2001; Saba et al., 2005; Reig et al., 2007) that are expressed in both dI1i and dI1c. Gain-of-function experiments showed that Barhl1 overexpression results in ectopic expression of Robo3, Nrp2 and DCC, and promotes midline crossing (Kawauchi et al., 2010). Using a similar approach, it was shown that Barhl2 also promotes a commissural phenotype and that Barhl2 overexpression leads to the induction of the adhesion molecule Tag-1 (Saba et al., 2003).

On the other hand, it has been reported that Atoh1 induces the expression of the LIM homeodomain TFs Lhx2 and Lhx9, either directly or indirectly through Barhl TFs (Bermingham et al., 2001; Gowan et al., 2001; Nakada, 2004). Barhl2 mutant mice exhibited a shift in the position of dI1i neurons from lateral to medial regions concomitant with a dramatic loss of ipsilateral projections and an increased number of commissural axons, which agrees with the observed aberrant upregulation of the homeodomain TF Lhx2 in the dI1i neurons of these mice. *In vitro*, Barhl2 binds to the regulatory sequences of Lhx2 and represses its expression (Ding et al., 2012). Gain-of-function experiments have shown that this TF is able to induce Lhx2 and another member of the same family, Lhx9 in spinal neurons (Kawauchi et al., 2010). Together these results suggest that Barhl2 represses Lhx2 in dI1i neurons to block the commissural phenotype. Independent gain-of-function experiments in the chick spinal cord suggested a role for Lhx9 in dI1c axons after midline crossing in the control of rostral turning and the dorsoventral positioning of axons in the longitudinal plane (Avraham et al., 2009), but these two Lhx factors seem to contribute to the diversification of dI1c and dI1i subtypes at earlier stages of dl1 differentiation.

Both Lhx2 and Lhx9 are expressed in dI1c neurons whereas dI1i neurons express only Lhx9 (Wilson et al., 2008). Single Lhx2 or Lhx9 mutant mice do not exhibit guidance phenotypes in dI1 neurons but commissural axons do not cross the midline in double Lhx2/9 mutants, similarly to the phenotype observed in Robo3 mutants (Sabatier et al., 2004). This pointed at Robo3 as a downstream target of Lhx TFs (Wilson et al., 2008). Chromatin immunoprecipitation (ChIP) assays *in vitro* and *in vivo* revealed that Lhx2 binds the Robo3 promoter (Wilson et al., 2008; Marcos-Mondéjar et al., 2012) and gain-of-function experiments in the mouse spinal cord demonstrated that Lhx2 is capable of inducing Robo3 (Kawauchi et al., 2010). All these experiments suggested that the Lhx2/9-Robo3 cascade is the default program in dl1 neurons and this program needs to be repressed in order to generate ipsilateral neurons. Supporting this idea, it was shown that ectopic expression of Robo3 in dorsal spinal neurons redirects ipsilateral axons towards and across the floor plate (Escalante et al., 2013). Interestingily, another member of the Robo family, Robo2, is differentially expressed in the dl1 subpopulations and, while dI1c projections are not affected in Robo2 mutants, dl1i axons project aberrantly through the motor neuron pool closer to the midline (Wurmser et al., 2021). Additionally, different components of the Wnt signaling pathway, including β-catenin and several Wnt receptors seem to be also required for midline crossing in dl1c (Avilés and Stoeckli, 2016).

Despite all this progress, it is difficult to reconcile a simple linear cascade in the gene regulatory network (GRN) specifying a commissural versus ipsilateral choice in dI1 neurons given the complexity of the regulatory mechanisms linking Atoh1, Barhl1/2, Lhx2/9 and downstream targets. Together with a more precise definition of the GRN controlling the specification of dl1 subtypes, other questions such as whether Lhx TFs activate other guidance receptors such as DCC, Robo2 or members of the Wnt pathway, or whether Robo3 expression is regulated by other homeodomain TFs in different types of commissural interneurons remain to be answered.

In the mouse visual system, the majority of retinal ganglion cell axons cross the ventral diencephalon at the optic chiasm level (cRGCs) while a minority of these axons project to the ipsilateral hemisphere (iRGCs). In this model, also largely used to study axon guidance mechanisms, another member of the LIM homeodomain TF family, Islet2 (Isl2), is differentially expressed in the ipsi and the contralateral RGCs subpopulations (Pak et al., 2004). Isl2 mutant mice show an increased number of iRGCs at the expense of the cRGCs. However, this only affects the subgroup of cRGCs that are born in the ventrotemporal region of the retina at late developmental stages and the targets of Isl2 to control the projection of this late-born RGC population have not been identified. The TF Pou4f1 (aka Brn3a) is also expressed in cRGCs but not iRGCs (Quina et al., 2005) but its function in axon guidance at the midline is still a matter of investigation. Finally, other TFs implicated in the establishment of cRGCs identity are the members of the SoxC family, particularly Sox4, Sox11 and Sox12 (Kuwajima et al., 2017). SoxC proteins bind to the Hes5 promoter to repress Notch signaling and induce cRGCs differentiation. SoxC genes regulate the expression of Plexin-A1 and Nr-Cam, which are required in cRGCs for correct axonal decussation at the chiasm (Kuwajima et al., 2012). Also, an ectopic ipsilateral projection is apparent in Sox4/Sox11/Sox12 triple conditional mutant mice (Kuwajima et al., 2017), suggesting that these proteins may be repressing the differentiation of iRGCs.

### Transcriptional Regulation of Axon Midline Avoidance

**FIGURE 1:**
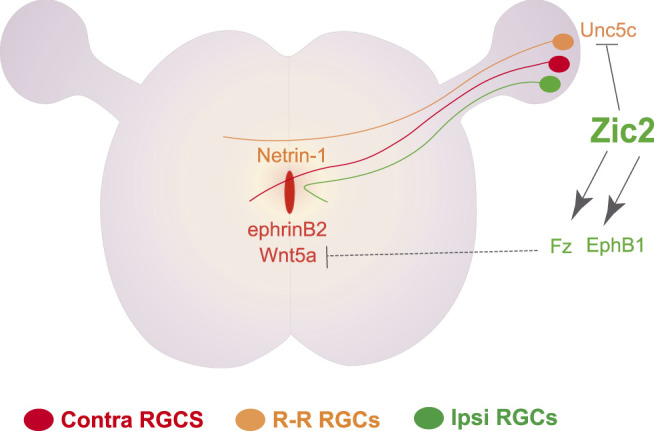
Transcriptional control of ipsilateral trajectories in the visual system. The TF Zic2 controls the trajectory of ipsilateral RGC axons through the transcriptional upregulation of the tyrosine receptor EphB1, which in turn mediates repulsion from glial cells at the midline that express ephrinB2. Concomitantly, Zic2 induces the expression of several Wnt receptors and cytoplasmic proteins to inhibit the attractive response towards Wnt5a -also expressed at the midline-experienced by contralateral RGCs. Zic2 also represses the expression of the Netrin1 receptor Unc5c in iRGCs to allow axon growth through a Netrin1-expressing area at the chiasm.

While the transcriptional regulation of midline crossing was originally described in dl1 spinal neurons, the regulation of axon midline avoidance was initially characterized in the visual system. The zinc finger TF Zic2, expressed in ipsilateral but not in contralateral RGCs, was reported as the main determinant of iRGC (Herrera et al., 2003). The expression of Zic2 and the generation of iRGCs in the ventrotemporal retina depends, at least partially, on the expression of CyclinD2 in a populatin of neural progenitors located at the ciliary margin zone of the embryonic retina (Marcucci et al., 2016). Functional experiments in mice initially demonstrated that Zic2 is necessary and sufficient to induce the expression of the tyrosine kinase receptor EphB1 that mediates axonal repulsion throught its ligand ephrinB2 expressed by midline cells (Williams et al., 2003; García-Frigola et al., 2008). The upregulation of EphB1 by Zic2 in iRGCs was later confirmed by chromatin immunoprecipitation assays followed by massive secuencing (ChIP-seq) which also identified other Zic2 targets including different members of the Wnt signaling pathway (Morenilla-Palao et al., 2020). In agreement with previous observations in spinal dl1c neurons (Avilés and Stoeckli, 2016), loss-of-function experiments in RGCs demonstrated that β-catenin is essential also for midline crossing in visual axons. Further functional experiments ruled out the canonical Wnt pathway as a regulator of axon guidance at the midline and demonstrated that, while contralateral axons enhance their growth upon Wnt5a exposure, ipsilateral axons collapse in response to Wnt5a, suggesting that a non-canonical Wnt signaling pathway mediates midline crossing. ChIP-Seq assays in RGCs also demonstrated that the differential response of ipsi- and contralateral visual axons to Wnt5a is regulated by binding of Zic2 to the regulatory regions of specific Wnt receptors and other Wnt signaling components such as Apc2. The induction of Zic2 results in the accumulation of β-catenin which is potentially phosphorylated by EphB1 at the growth cone after contact with ephrinB2 at the midline (Morenilla-Palao et al., 2020). Another component of the Zic2-controlled program is the Netrin receptor Unc5c. Unc5c is expressed in a subset of cRGCs that transiently project to the opposite retina during early postnatal stages. Netrin1 is expressed in the ventral diencephalon to impede the growth of these retino-retinal axons into the optic chiasm. In iRGCs, Zic2 binds to regulatory regions near the *Unc5c* locus and represses its expression in order to facilitate their growth into the diencephalic region (Murcia-Belmonte et al., 2019) (Figure 1). Thus, Zic2 binds to the regulatory regions of many genes, including EphB1, different components of the Wnt pathway and Unc5c, to specify iRGCs and regulate their guidance at the midline.

The positive correlation between the number of ipsilateral axons and the expression of Zic2 in the retina of different species pointed to this TF as a determinant of iRGCs identity across evolution. In addition to being expressed in the developing mouse retina, Zic2 is expressed in ferrets in a larger retinal area that coincides with the zone occupied by iRGCs in this species. In humans, Zic2 and EphB1 are both expressed in the temporal half of the retina also coinciding with the location of iRGCs and, in *Xenopus*, Zic2 is expressed in the retina during metamorphosis when a late-born ipsilateral projection is generated. However, in zebrafish and chicken Zic2 is not expressed in RGCs during development and accordingly these species lack an ipsilateral projection (Herrera et al., 2003; Lambot et al., 2005; Murcia-Belmonte et al., 2019; Vigouroux et al., 2021). Interestingly, ectopic expression of Zic2 in zebrafish RGCs leads to the appearance of an ectopic ipsilateral projection (Vigouroux et al., 2021), revealing that Zic2 is able to activate a transcriptional module that controls midline avoidance even in species that naturally lack an ipsilateral projection. Recent reports have shown that non-teleost bony fish also have an ipsilateral retinal projection (Vigouroux et al., 2021) but the function of this projection is still unknown and future experiments are needed to uncover this question and also to dilucidate the regulatory mechanisms that control this ancient ipsilateral projection.

**FIGURE 2:**
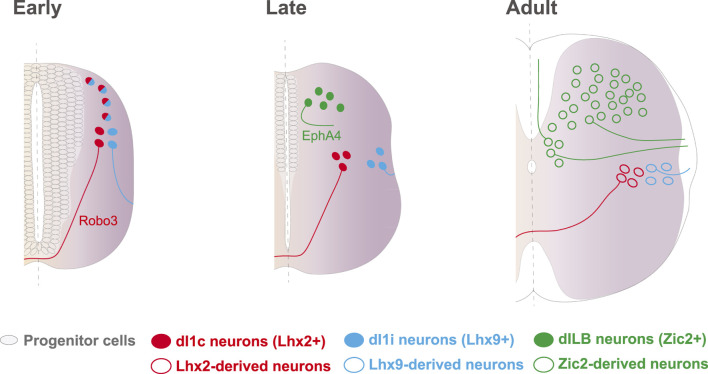
Axon guidance strategies in different subtypes of spinal cord neurons. dI1 neurons are born early in neural tube development and are separated from the midline by the subventricular zone, populated by progenitor cells. Expression of Lhx2 in dl1 neurons activates a contralateral program, in part through the upregulation of Robo3. Ipsilateral dI1 neurons do never confront midline cues and project their axons into the ipsilateral lateral funiculus, likely through the expression of Robo2 and possibly mediated by Lhx9. Later, by the time that dILB neurons are born, progenitor neurons have already differentiated and postmitotic neurons distribute at both sides of the dorsal midline. In this scenario, EphA4 and likely other guidance molecules, are controlled by Zic2 to ensure midline repulsion and ipsilateral projection through the dorsal and lateral funiculi. These TFs are downregulated following development and are not expressed in adulthood.

Further functional experiments in chick and mice demonstrated that Zic2 does not only determines axon midline avoidance in the visual system but also in other types of ipsilateral neurons such as the thalamocortical projections and the late-born population of excitatory interneurons (dILB) located in the dorsal horns of the spinal cord. dILB neurons are born very close to the dorsal midline (Alaynick et al., 2011; Gross et al., 2002; Helms and Johnson, 2003; Lewis, 2006; Müller et al., 2002; Petkó and Antal, 2012). These cells but not their inhibitory counterpart dILA neurons that project locally and contralaterally (Escalante and Klein, 2020; Tulloch et al., 2019), express Zic2 which, in turn, is necessary and sufficient to define their ipsilateral trajectory (Escalante et al., 2013) (Figure 2). Chromatin immunoprecipitation experiments in a cell line and in spinal neurons, demonstrated that Zic2 is able to bind to the promoter of another Eph receptor, EphA4. Further functional experiments in chick and mice also confirmed that, instead of regulating EphB1 as in the visual system, in spinal neurons Zic2 controls the expression of EphA4 (Escalante et al., 2013; Luo et al., 2015; Morenilla-Palao et al., 2019). As EphB1, EphA4 binds to ephrinBs to mediate axon repulsion and it has been shown that ephrinB1, ephrinB2 and ephrinB3 are all expressed at the spinal cord midline (Kullander et al., 2001; Kullander et al., 2003; Escalante et al., 2013; Paixão et al., 2013; Klein and Kania, 2014; Haimson et al., 2021).

All together, these observations point to the existence of several gene programs that control axonal laterality in ipsilateral spinal neuron populations with dispar ontogeny. Early born dl1 neurons locate far away from the midline because the ventricle and the subventricular zone (SVZ), which is rich in progenitor cells, occupy the medial region of the dorsal tube. As progenitors exit the cell cycle, the SVZ shrinks and the somas of the late born dILB neurons locate close to the midline. In contrast to the dl1i population whose axons never approach the midline and their projection patterns rely on Lhx factors, dILB neurons are born in close contact with the midline and their axons need to be repelled as soon as they start growing in order to project ipsilaterally. Thus, it is not surprising that although both populations, dI1i and dILB neurons project ipsilaterally, they developed alternative strategies to control the guidance of their respective axons (Escalante et al., 2013) (Figure 2).

## Conclusion

Despite the increasing number of rapidly emerging innovative techniques that largely facilitates research on the transcriptional mechanisms regulating gene expression, only a handful of TFs have been convincingly shown to control genetic programs involved in the regulation of axonal behaviors. In the last decade, the interest to understand how neural circuits function has exponentially increased and the development and application of genetically encoded, magnetic and thermal tools to manipulate neuronal circuits is helping us to disentangle brain connectivity and circuits function. However, it is surprising that in the era of next generation sequencing and single cell transcriptomic approaches (Escalante et al., 2020) there are still very few studies taking advantage of these technologies to elucidate the genetic programs that precisely control the definition of axonal trajectories. Incorrect circuit wiring during embryonic development may have a huge impact in the adult individual and we are still far from understanding how circuits are built in the first place. Future efforts devoted to understand the regulatory logic underlying neuronal trajectories will certainly contribute to prevent pathologies derived from neural circuits miswiring.
